# A Case of Hydrophobia

**Published:** 1809-04

**Authors:** G. Borrett

**Affiliations:** Yarmouth, Norfolk


### A CASE OF HYDROPHOBIA.

IN the evening of January 2, 1809, a dog, from a neighbouring village, affected with *rabies canina*, entered this town, and bit several people, not less than ten, one of whom was the unfortunate Elizabeth Kirk, aged 29 years. Cattle in the neighbouring villages were also bitten, and some pigs in the environs of Yarmouth. Elizabeth Kirk had several wounds on the right hand, ring finger of the same hand, and on both arms.

Mr. Taylor, the medical gentleman to whom she applied, cauterized the wounds, and kept up a discharge from them by blistering ointment; but from the situation of those in the hand, it was to be apprehended that neither the power of the caustic, nor excision, could have acted deeply enough to insure her safety, without, at the same time, injuring the use of the hand.

The wounds had healed about ten days before her indisposition, which began on Monday, February 12, forty-one days from the accident, by pricking sensations, which continued twenty-four hours in the bitten parts, and they were tender to the touch.* Some of the cicatrices were hard and inflamed, and particularly that on the ring finger, and another between the metacarpal bones of the ring and little fingers of the right hand. She did not acquaint Mr. Taylor of her illness until the Monday evening, six o'clock, when she gave the above description of the uneasiness in the wounds; seemed much dejected, complained of great lassitude and thirst, accompanied with pyrexia, and for some time drank freely and repeatedly of liquids, particularly of water. Mr. Taylor prescribed a blister between her shoulders, and a mixture with tinct. opii and mist. camphorat. of which she took several doses.

Tuesday morning, ten o'clock, Mr. Taylor called a consultation of all the physicians and surgeons of the town, including those of the Naval Hospital, when she gave the following description of her feelings. The pricking uneasiness of the wounds extended up the right arm, in the course of the lymphatics to the axilla, across the chest and mammæ; and as this symptom subsided, she felt great anxiety about the præcordia, attended with a particular constriction around the throat, and pain at the cartilago ensiformis. Her eyes and whole countenance were expressive of great and anxious lassitude, and she sighed constantly.

* One of her acquaintances says, she felt uneasiness in the bitten parts from the Friday preceding.

stantly. She was timid, and all the external senses were more acute than they naturally are. She could neither bear the moving of your fingers to her throat, nor the agitation of the air in the room, without anhelatio. She now expressed much dislike to the swallowing of any liquid; but from our intreaties she made several efforts to do it, which were violent and convulsive, and indicated great pain, her eyes darting such an expression of anxiety and distress as is not to be easily described. Her tongue was covered with a thin white fur; the pulse was small and slow, not more than 70 in a minute; her extremities were cold, with a state of body approaching to syncope, and a constant extrication of wind from the stomach by a convulsive hiccough. This dyspeptic state of the stomach or singultus, was particularly impressive to the observers, and distressing to the patient.

Her bowels being costive, the medicines first prescribed in consultation were pulv. jalapii ℈ij. calomel. ppt. ℈j. muc. q. s. f. bol. statim s. and broth clysters. A blister to the epigastric region, and another to the throat; and as soon as the bowels should be cleared, a pill containing an eighth of a grain of arsenic with crumbs of bread, every two hours.

Tuesday evening, nine o'clock, She had had three copious stools from the bolus and enema, and had swallowed two arsenical pills. The blister upon her throat she would not suffer to remain, because it increased the suffocating constriction. She entreated us not to ask her to swallow any fluid, as the attempt occasioned her great pain and spasm. She seemed more tranquil, except when the convulsions came on, which were now very violent. She was perfectly collected between the fits, and seemed only apprehensive we should touch her neck or face, which produced great distress to her; her pulse was very feeble but regular, the heat of the body much below natural.

At two o'clock on Wednesday morning, I saw her with Mr. Taylor; the convulsions were very violent, the pulse was irregular and intermitting, the pain about the epigastric region very great, and the appearance of distress and horror much increased. There was a cold dew upon her extremities, and the pain of swallowing was insupportable. At this time we prescribed pills containing one grain of opium and five of camphor, to be got down frequently.

At ten o'clock on Wednesday morning, the medical gentlemen met, to see again this shocking victim of rabies canina, whose sufferings now appeared drawing to an end. She had not been able to swallow any of the pills, or any liquid since we left her. She lay upon her back; her face looked purple and rather bloated; the pulse had ceased to beat at the wrist; she was constantly ejecting from her mouth viscid saliva, which run down her chin, upon her breast, but she could not bear it to be wiped off, for even the touching of her face excited *convulsions*; and if by accident the attendants who held her, let a lock of her hair fall upon her face, *these* followed. Her skin had a clammy cold feel, and her eyes and countenance expressed the greatest conceivable distress. The horror of this last scene is not to be described, those only who have witnessed this dreadful malady can have an idea of it. At about eleven o'clock the convulsions ceased, she seemed calm, and knew her friends, as she had done throughout the disease. At half past eleven she became insensible, and expired at twelve at noon.

APPEARANCES ON DISSECTION OF THE BODY,
Which took place at eight o'clock on Thursday morning, twenty hours after death.

On opening the head, the dura mater was in a natural state. The vessels of the pia mater were preternaturally distended, and exhibited unequivocal appearances of turgescence, greater even, I believe, than the generally thought turgid representation of the brain by Mr. Charles Bell, and between the pia mater and tunica arachnoides, there was general watery effusion covering the lobes of the cerebrum. The brain was firm, and bore the knife well, and had a healthy aspect. The vessels of the plexus choroides were very turgid, and the lateral ventricles contained about two drachms of clear fluid, whilst the third and fourth were natural. A considerable quantity of fluid appeared about the sella turcica, (from the position of the head in making the dissection); and when the lobes of the cerebellum were removed from the basis of the skull, there seemed about half an ounce of fluid. The longitudinal and lateral sinuses were empty.

Thorax.—The right lung was sound, the left from previous disease, adhered to the pleura throughout its whole extent, was completely collapsed, dense, and of a redder colour than usual, and appeared impervious to air. The heart natural, and its pericardium contained about half an ounce of fluid; the ascending and decending cava gorged with blood.

Upon opening the abdomen, the liver appeared in a perfectly healthy state, and the gall-bladder nearly full of a well coloured bile. We were struck with the distension of the stomach and small intestines by flatus. The intestinal canal contained little fæcal matter, and the cæcum, colon, and rectum were not at all distended. The stomach and the greater part of the duodenum, with the œsophagus, pharynx and tongue, and the larynx with the trachea to its bifurcation, were removed from the body to an apartment more convenient for accurate observation. The œsophagus was then slit open at its posterior part, (where it is opposed to the vertebra) and the incision continued along the greater curvature of the stomach, through the pyloris and duodenum, and the parts lightly rinced in cold water. The stomach contained about ℥iv. of bilious fluid, and its whole villous coat was highly tinged with bile. The tongue was covered with a thin layer of white fur, and the mucus glands at its basis were much enlarged. The upper part of the œsophagus appeared free from inflammation, and covered with its fine cuticle, till within *four inches of the cardia, where there were evident marks of inflammation*; the cuticular covering of the œsophagus was seen at this part, terminating as it were *by a loose frittered edge; and from this point to where the œsophagus expands into the cardiac orifice of the stomach, the cuticle was wholly gone*; the œsophagus *at the cardia, and for two inches upwards, shew strong marks of having undergone great inflammation, the cuticle was completely destroyed; the mucus membrane had passed through the first stage of inflammation, had lost its redness, and assumed an ash colour, with dark coloured patches; the cardia had the same dark coloured spotted appearance,* and the inflammation extended to nearly the *breadth of the palm of a hand on the upper orifice and larger end of the stomach,* which had a studded appearance of red points, these points becoming fainter as the inflammation extended in the direction of the larger curvature; in appearance, the redness was not unlike a well injected maternal part of the placenta; the rest of the stomach appeared free from inflammation.

The larynx was natural; neither glottis nor epiglottis were inflamed, nor any part of the trachea. A small quantity of mucus lay loose in the pomum Adami.

However sceptical some men may have been, or still are, as to the existence of the disease called Hydrophobia, I will venture to assert, that the symptoms have been so accurately detailed in various publications within the last two years, that any one possessed of the commonest attention to the phænomena of disease cannot fail to recognize this direful malady at the first glance, and to do immediate justice to the veracity and discrimination of the many respectable practitioners who have lately written on this subject.

It will be difficult to admit Dr. Mosely's Case; every medical man knows how to appreciate mercury, a medicine whose powers we have looked for more than those of any other over this disease, and have been as constantly disappointed; although no one will deny, that the smart salivation and corporeal punishment inflicted in *this case of violent hysteria from terror*, restored the lady to her senses.

In the Medical and Physical Journal for March 1807, page 271, of the xviith vol. a Case of recovery from Hydrophobia is narrated by Mr. GEO. HICKS of Baldock. Any one who will examine this case, must doubt its being a true case of hydrophobia, as produced by the poison of a rabid animal, and to justify Mr. WARD's judicious Observations upon it in May following, vol. xvii, p. 457. I was surprised to see at the end of Dr. WOOD's excellent Observations on the Symptoms of Hydrophobia, in the Medical and Physical Journal for February 1809, the Case of Robert Dixon selected and brought forward again to the public, as an instance of recovery, which, it is said, was effected by antispasmodic medicines, opium, musk, &c. &c. and a warm antispasmodic plaister to his throat. Whoever will take the trouble to refer to this Case, given by Mr. Wm. TURNBULL, Warwick Court, Holborn, in the Medical and Physical Journal for February 1808, page 118, vol. xix. will suspect it to be a case without any pathognomonic symptoms of hydrophobia, but originating in terror, and ending in hysteria. Many other cases might be selected of doubtful character: So may cases of tetanus be adduced, following punctured wounds some time after the accident, having symptoms of hydrophobia; but in these, the continued stiffness occasioned by the spasms on the muscles, keeps the jaws closed and fixed, which is essential to tetanus, and will at once lead to a distinction of the disease independently of the circumstance of infection, which must always be connected with true Hydrophobia.

HAMILTON on Hydrophobia, vol. i. page 84. "Hydrophobia, we have admitted, exerts its violence upon the nerves. Every symptom observable in this disorder serves as a proof. It is for this reason we must contradict the illustrious Boerhaave, in his denominating it summè inflammatoricus, as neither the dissections, nor other phenomena authorize the assertion. It is true, there is a species of inflammation which dissection detects, observable on the inner surface of the lower part of the œsophagus, and over the internal superfices of the stomach."* In the next stage, he observes, "Dr. Ferriar, in a late inspection, has evidently proved the existence of these abrasions; and from the short distance between death and his examination, we may rely on these appearances as absolutely belonging to the disease, and not to changes from putrefaction after the extinction of life." In vol. ii. page 381, is the account of the dissection of Dr. Ferriar's second case, twenty-four hours after death. "Internally; head, effusion of fluid between the pia mater and tunica arachnoides, considerably distending the former, &c.—Thorax, some adhesions of the pleura, &c. The left lobe so completely filled with blood, as to have acquired considerable weight and solidity.—Abdomen, liver changed in colour, &c.—Stomach, *much inflamed, especially on the great curvature.* Villous coat inflamed in irregular points, with an appearance of abrasion, similar to those found in the Doctor's former patient.—Pylorus sound; œsophagus sound." "From the above appearances, Dr. Ferriar is inclined, contrary to his former opinion, to consider the disease as of an *high inflammatory nature*, and analogous to peripneumonia."

Dr. VAUGHAN'S Case, vol. ii. p. 433, on dissection,—"Stomach, marks of inflammation, with a small quantity of fluid mixed with the medicines."

In Mr. ASTLEY COOPER'S Communication to Dr. Babington,* p. 555, Hamilton, vol. ii. "The appearances on dissection (of a rabid dog) observed by Mr. Cooper, were, an inflammation of the internal membrane of the stomach, with several small clots of blood effused both within the stomach and in the cellular substance connecting the mucous coat to the muscular. A large circular spot of inflammation was discovered in the pharynx. Mr. C. has met with the same appearances in several other dogs; hence the conclusion formerly drawn is strengthened, that the stomach, as well in the canine as the human tribe, is much connected with, if not the principal seat of the disease." †

The cases given by Dr. BEDDOES, in the Medical and Physical Journal for September 1808, vol. xx. page 195, are worthy attention; as is also that by Dr. POWELL, which follows, page 201, with the appearances on dissection. "The vessels of the brain and its membranes were much loaded with blood, and nearly half an ounce of colourless fluid was contained in the ventricles. The intestines were much distended, &c. The œsophagus was con-

* Vide Medical Records and Researches, &c. p. 136.

† Vide Baillie's Morbid Anatomy, p. 141, on the appearances of the stomach in Hydrophobia.

tracted, and strongly marked with longitudinal rugæ; it was somewhat redder than natural. The trachea was in a natural state. The stomach, &c. upon its internal surface, which was rather redder and more vascular than natural, there were, under the internal membrane, some small spots of extravasated blood collected into clusters, and were chiefly found about the cardia."

The excellent and accurately described case of Doctor Pinckard, in the Medical and Physical Journal for Janurary, 1809, and the appearances on dissection, twenty-nine hours after death, seem to merit the particular attention of medical gentlemen. In this he describes the whole of the pharynx and œsophagus as in a state of inflammation; and says, "At the lower part of the œsophagus, about half an inch from the cardiac orifice of the stomach, was an eroded spot, nearly the size of a shilling, assuming an appearance as if the inner coat had been separated and shrivelled up by scorching. The stomach and intestines were much distended with flatus, &c. A few inches below the cardia was a fullness of the vessels of the villous coat, which caused a spotted and circumscribed redness about three or four inches in diameter.

The concurring testimony of all these detailed cases of dissection is strongly and unequivocally established by the examination of Eliz. Kirk's body after death, and must bring conviction to the mind of every man of the parts most affected, and will I trust establish, beyond all doubt, the true nature of this disease, as depending upon inflammation of the cardia, and of the cardiac portion of the œsophagus; and that, for any rational hopes of cure, we must pursue the same active antiphlogistic treatment as in Gastritis, to which it bears stronger resemblance than to any other disease. The morbid sensibility or excessive irritability of the skin of a hydrophobic person is peculiar to the malady, and distinguishes it equally from Tetanus, to which it has been also compared.

That inflammation of this part of the œsophagus and cardia really takes place in all cases of *true* hydrophobia, I think there is no doubt from the above cases; and the dissection of a pig at the Barracks, bitten at the same time, and by the same dog, also clearly proved it. The pig shewed signs of indisposition on the *ninth* day after the bite, refused his food on the tenth, and became convulsed and died on the thirteenth, the fourth day of the disease. Upon opening the œsophagus and stomach, the same parts exhibited the same appearances as the human stomach, but less in degree; the stomach was tinged with bile, and a small quantity of mucus was formed about the head of the trachea.

It must be evident to every one, that the disease called Hydrophobia, is, in the patient, not a dread of water, any more than of any other fluid, *but a dread of swallowing*, arising evidently out of the state of the parts diseased by inflammation.*

It will be seen, that as soon as the pricking sensations ceased in the bitten parts, anxiety about the præcordia and pain at the cartilago ensiformis began, followed by a dyspeptic state of the stomach and singultus, with pain in swallowing. She had drank water eagerly during the pyrexial or first stage of the disease, but now (10 o'clock Tuesday morning) she expressed great pain in swallowing, more from liquids than solids, because to swallow a liquid, a greater contraction of the muscles of deglutition is required than for a solid; which contraction, by putting the œsophagus into a state of greater tension, produces greater pain and spasm. In a few hours, as the inflammation increased, she could swallow neither liquid nor solid, and the alternate actions of contraction and relaxation of the œsophagus seemed altogether suspended, as was proved by the constant ejection of the saliva. Dr. Pinckard's patient being able to swallow water, and for which he was so anxious during the latter moments of his existence, I conceive may be accounted for, from the lower part of the œsophagus and cardia having lost their vitality and undergone such a change of organization, as that the act of swallowing no longer produced pain; and, like the fatal ease and tranquillity in enteritis, proclaimed the approach of death.

From the extreme sensibility of the stomach it is called the grand sympathizer of the body; the cardia of this *viscus*, and the cardiac portion of the œsophagus, we have seen to be the immediate seat of hydrophobic inflammation, which will, upon physiological analogy, readily account for the contraction and spasm of the pharynx. We know that any indigested food approaching the pyloric orifice of the stomach, stimulates it to contract, and sets up a retroverted action to a certain degree, in order to prevent the escape of this unassimilated matter into the duodenum. This premonitory case is duly and nicely observed until the food is dissolved, or that from its acrimonious stimulus, the natural action of the stomach is completely inverted, and vomiting follows: may not then, great

* It is a well known fact, that dogs, under Rabies Canina, readily lap water but do not swallow it, &c.—Barker Daniel, p. 138, vol. 1. 1805.

excitement of the cardia and cardiac portion of the œsophagus produce an irregular and spasmodic action or sympathy of the pharynx? The idea of this sympathetic effect will be still more supported, if we bring to our recollection, that the œsophagus and stomach are supplied with nerves principally from the eighth pair, or par vagum; nor is it more unphysiological to admit the spasm at the *upper* end of a membranous tube like the œsophagus, from irritation at its *lower*, than the sympathy which is referred to the extremity of the urethra, from a stone irritating the membrane of the bladder; or in cardialgia, the sensation of heat and unneasiness being felt in the pharynx.

The inflammation of the œsophagus stimulating the mucus and salivary glands, accounts also for the great flow of saliva during the last eight or twelve hours of the patient's existence, which cannot be explained upon the supposition of hydrophobia being a tetanic disease, there being in tetanus no increased secretion of saliva; nor has any one medicine, upon this theory of the complaint, afforded the most trifling relief.

If we compare hydrophobia, as marked in this case, and in Dr. Pinckard's, with the symptoms of gastritis, we shall be struck with the analogy of the diseases. Cullen says, "Gastritis is known by an acute pain in some part of the region of the stomach, attended with pyrexia, with frequent vomiting, especially upon occasion of any thing being taken down into the stomach, and frequently with hiccough. The pulse is commonly small and hard, and there is a greater loss of strength in all the functions of the body, than in the case of almost any other inflammation." To this description may be added, extreme anxiety; an almost continual painful hiccough; the pulse is generally more sunk than in other inflammations of the viscera. "Sometimes a temporary madness ensues; and there is an instance in the Edinburgh Medical Essays, of gastritis being attended with a hydrophobia."

The disease called hydrophobia, or inflammation of the cardia of the stomach and cardiac portion of the œsophagus, begins with a pricking uneasiness in the bitten parts; is followed by pyrexia and thirst; great heat and pain about the epigastric region, and constriction about the pharynx; great anxiety, and constant eructation of flatus; deglutition very painful, and attended with convulsions, and the complaint marked by *excessive irritability*; the tongue covered with a thin white fur; the pulse soon becomes small and slow; a coldness of the extremities, and a state of body approaching to syncope, succeeds, with constant hiccough, and at length the pain of swallowing is insupportable, and the convulsions dreadful.

By this it will be seen that the two diseases agree in their leading features; which are, great pain about the epigastric region, attended with pyrexia; great anxiety at the præcordia, eructations, and hiccough; the pulse small and slow, (sometimes hard); great loss of strength, approaching to syncope, with painful deglutition. The pulse particularly marks the character of the two diseases, which is more feeble and slow than in enteritis, intermitting, and exhibiting the same phœnomena as in that disease.

In slight blows on the stomach the pulse ceases for a time, and not unfrequently in these accidents entirely; such is the sensibility of that organ and its connexion with vitality, that it may be said to be the seat of sensibility, as the brain is of sensation.

Under this view of Hydrophobia, we see why opium, musk, camphor, and the whole list of antispasmodic medicines have failed, and must ever fail of relief; why mercury, arsenic,* and the cold bath, are followed with no better success, and every other means which is not directed to diminish immediately the diseased excitement about the cardia and œsophagus. This end can be effected only by large and repeated bleedings even to syncope; and so early as the pricking sensatious in the bitten parts spread to the throat and epigastric region, to which a large blister should be applied. Clysters of broth and gruel are useful. No medicine should be exhibited by the mouth, unless any one can be found which has a directly sedative effect on the stomach. Will not the first stimulant effects of digitalis, aconite, henbane, or cicuta, on that organ, do more injury than their consequent sedative effects on the heart can do good? The very act of swallowing must be injurious, and to enforce it, cruel; but if any thing must be swallowed, barley water, or the common almond emulsion appears to me the most proper drink; but the patient should be left to please himself.

It may be said, that death does not take place in hydrophobia from inflammation. I would ask, from what then does it take place? If from excessive irritability, why have not the many and various antispasmodic medicines afforded relief?

---

* Arsenic has been particularly recommended from its being a well known and principal ingredient in the Tanjore pill, celebrated for the cure of the bites of serpents in the East-Indies; but the analogy does not hold good in the bite of the rabid animal of this country. The serpent secretes a poison for its defence, bland and innoxious to itself; whereas the poison of a rabid animal is deleterious to itself, and destroys the animal which secretes it: in course the two poisons are dissimilar, and are governed by their peculiar laws.

Supported by a physiological and pathological induction of facts, we are led to hope, that the same ratio of success may attend prompt and well directed efforts in this dreadful disease, as in the cure of gastritis, unless the inflammation in hydrophobia produced by an animal poison, sui generis, differs in all its laws from all other inflammations of that organ, and defies the control of art.

It may be urged that bleeding and the antiphlogistic treatment has been repeatedly tried both by the ancients and moderns without success; but in answer, I would beg leave to observe, so far as I am able to learn, that the bleedings have been too late, for surely we can no more expect to cure hydrophobic inflammation of the stomach, &c. by late bleedings, than we can expect to cure gastritis, arising from any other cause by bleeding, when the disease is far advanced. Should our best endeavours be unsuccessful, the state of a patient quiet and composed, sinking into the arms of death, under the effects of the lancet, will I think, be at least a spectacle less horrid to contemplate, than the necessary termination by dreadful and repeated convulsions.

The preventive means to be relied upon, are well understood by every practitioner; *complete and early excision of the bitten parts,* then ablution with cold water till the bleeding ceases, and the application of the kali purum to the whole extent of the wound, and even some lines on the surrounding skin. Where excision cannot with safety be performed, ablution with cold water should be long continued, and the kali purum freely used. Where these necessary steps have been neglected, no period before Hydrophobia takes place should be thought too late for the foregoing practice.

G. BORRETT.

*Yarmouth, Norfolk, March* 14, 1809.

